# Avaliação de dor do recém-nascido durante punção arterial: estudo observacional analítico

**DOI:** 10.5935/0103-507X.20210058

**Published:** 2021

**Authors:** Rayanne Marques Costa Alberice, Silvia Cristina Oliveira da Silva, Anna Caroline Costa Leite, Bruna Figueiredo Manzo, Delma Aurélia da Silva Simão, Juliana de Oliveira Marcatto

**Affiliations:** 1 Escola de Enfermagem, Universidade Federal de Minas Gerais - Belo Horizonte (MG), Brasil.

**Keywords:** Dor, Medição da dor, Enfermagem neonatal, Recém-nascido, Punções, Unidades de terapia intensiva neonatal

## Abstract

**Objetivo:**

Avaliar a intensidade de dor durante a punção arterial realizada em recém-nascidos internados em uma unidade de cuidados progressivos neonatais e avaliar a percepção do profissional em relação à dor neonatal.

**Métodos:**

Estudo observacional analítico, em que foram observadas 62 punções arteriais realizadas em 35 neonatos. Avaliou-se a dor durante a coleta pela escala *Premature Infant Pain Profile*. Os profissionais responsáveis pela coleta avaliaram a dor pela escala numérica verbal de zero a dez. Os dados foram submetidos à análise estatística descritiva por meio do programa *Statistical Package for the Social Science*.

**Resultados:**

Entre os recém-nascidos, 30,6% (n = 19) não tiveram dor ou tiveram dor leve (0 - 6), 24,2% (n = 15) apresentaram dor leve a moderada (7 - 11) e 45,2% (28) dor intensa (12 - 21). Constatou-se que os profissionais identificam a dor durante o procedimento.

**Conclusão:**

A punção arterial é considerada um procedimento doloroso e pode resultar em dor leve a intensa, sendo necessária a adoção de estratégias sistematizadas de avaliação, possibilitando a intervenção terapêutica adequada.

## INTRODUCTION

In recent years, scientific advances and the incorporation of new technologies in the treatment of newborns (NB) admitted to neonatal progressive care units (NPCUs) have resulted in greater survival and higher quality of care provided to this population. As a result, numerous diagnostic and therapeutic procedures are performed daily in NB admitted to NPCU, potentially resulting in experiences classified as painful, very painful or stressful.^(1)^

The functional and neurochemical elements necessary for the transmission of painful impulses to the cerebral cortex are present in term and premature NB.^(2)^ The pain experienced in the neonatal period can result in short-, medium- and long-term complications because the conduction and interpretation of the pain process can generate physiological, structural and functional changes, in particular related to the central nervous system of NB. The immediate aggravations and clinical complications include periventricular hemorrhage and altered neurobehavioral responses. Regarding long-term complications, emotional and social interaction problems may occur, in addition to the development of psychopathologies in adolescence and adulthood.^(3)^

The nonverbalization of pain and accommodation to persistent pain are unique characteristics that hinder pain assessments in NB. However, assessments are essential for proper management and are performed in this age group by identifying physiological and behavioral changes.^(4)^ Thus, pain assessments are necessarily indirect and require trained professionals, in addition to validated methods applicable to clinical practice, to identify this clinical condition. According to a systematic review, approximately 65 scales can be used to assess pain and sedation in childhood. Such scales have undergone validation and reliability testing and consist of physiological and behavioral variables and are indicated for use in different contexts related to age and clinical condition.^(4,5)^

Despite the large number of available scales, few have been translated into Portuguese and culturally adapted to Brazil. The instruments most suitable for assessing pain in NB in Brazilian neonatal units are the Neonatal Infant Pain Scale (NIPS), the Neonatal Facial Coding System (NFCS), the Premature Infant Pain Profile (PIPP) and its revised version, the Preterm Infant Pain Profile-Revised.^(5)^

The PIPP is a mixed scale that assesses six indicators - alertness, maximum heart rate, minimum oxygen saturation, brow bulge, eye squeeze and nasolabial groove - differently based on gestational age, considering that the lower the gestational age, the lower the behavioral changes secondary to pain stimulation. The score ranges from zero to 21 points; scores less than or equal to six indicate an absence of pain; scores from seven to 12 indicate mild to moderate pain; and scores greater than 12 indicate the presence of moderate to severe pain.^(6)^ In the revised version, the use and scoring of the PIPP were made easier to improve its application in clinical practice.^(5-7)^

For decision making, it is extremely important to determine the real need and clinical relevance of a painful intervention when proposed, considering that nonexposure is the only truly safe way to avoid pain. Once the indication of a procedure is confirmed, when an intervention is known to be painful, the most appropriate therapeutic strategy should be indicated, and pain must be assessed during and after the procedure.^(8)^

Arterial puncture is a painful procedure often performed in the NPCU and consists of the collection of biological material for clinical and laboratory monitoring. Despite the advances that have occurred in the last two decades regarding the evaluation and treatment of pain in the neonatal period, pain resulting from arterial puncture has been little investigated among NB, as evidenced by the lack of adoption in clinical practice of the recommendations described in the literature.^(9-12)^

In this context, the present study evaluated the intensity of pain during arterial puncture performed in NB admitted to a NPU and evaluated the health professionals’ perception of neonatal pain.

## METHODS

This was an observational analytical study conducted in an NPCU of a public hospital in Belo Horizonte, Brazil, from June to September 2018. The inclusion criteria were NB admitted to the NPCU who underwent arterial puncture in the first 28 days of life, without the use of neuromuscular blockers, opioids or analgesics in the 48 hours prior to inclusion in the study. Newborns with a known diagnosis of neuromuscular dysfunction were excluded from the study. For patients who required more than two puncture attempts, only the first two experiences were included in the analysis, considering the pain accommodation process in situations of persistent pain.

Data were collected by four researchers previously trained in the use of the PIPP scale. Ten patients were evaluated under the supervision of the research coordinator, allowing alignment, clarification of doubts and analysis of agreement. In this phase, the evaluators observed the same patients, and because no changes were necessary in the research protocol, the evaluations performed in this phase were included in the final study sample.

Collections were made during day shifts, from Monday to Friday, at the standardized blood collection times in the NPCU. The data collection instrument was composed of two parts: the first for recording sociodemographic data of the sample (sex; gestational age; birth weight; Apgar score; diagnosis at birth; and maternal education) and the second for recording the procedure (pain scale score before, during and after; heart rate; oxygen saturation; and health professionals’ perception of the NB’s experience of pain). The data from the first two attempts to collect arterial blood were analyzed.

The arterial puncture procedure was divided into four time points (T1, T2, T3 and T4), with the objective of identifying pain resulting from manipulation and rupture of the skin integrity, as follows: T1 - baseline evaluation at 5 minutes before the procedure; T2 - degermation; T3 - puncture; and T4 - return to baseline 5 minutes after the end of the procedure.

The pain resulting from arterial puncture was assessed by the researchers using the PIPP at the four procedure time points.^(13)^ The assessment performed by the health professionals responsible for blood collection was based on their perception of pain experienced by the NB, using a verbal numerical scale ranging from zero to ten; the researchers considered a score of zero to three as mild pain, of four to seven as moderate pain, and of eight to ten as severe pain. The health professionals evaluated pain only once, at the end of the procedure.

The pain protocol in the study unit included the administration of oral 25% glucose solution 2 minutes before the procedure and facilitated restraint during arterial puncture for all NB.

The independent variables analyzed were corrected gestational age, birth weight, main diagnoses and health professional responsible for collection. The dependent variables were the number of punctures, duration of the procedure, PIPP score at the different procedure time points and perception of pain reported by the health professionals using the verbal numerical scale.

The guardians of the participating patients were informed of the nature of the study through an informed consent form. The study followed ethical precepts, in accordance with resolution 466/12, and was approved by the research ethics committee, with the consent of the institution in which it was conducted, under opinion n. 2.144.864 (CAAE 66157317.9.0000.5149).

Data were initially inputted in Microsoft Excel spreadsheets, version 2016, and then uploaded to the Statistical Package for the Social Sciences (SPSS) version 21.0 for Windows. The interrater agreement was analyzed during the pilot study using the kappa coefficient, which showed strong agreement (K = 0.76) among the researchers who performed the pain assessment using the PIPP. The variables were analyzed descriptively, adopting measures of central tendency, such as the mean, median, minimum, maximum and standard deviation for numerical variables and absolute and relative frequencies for categorical variables. Next, the Shapiro-Wilk and Kolmogorov-Smirnov normality tests were performed to investigate the distribution of the variables. The results revealed that the data had a nonnormal distribution, and thus, nonparametric tests were applied. The Wilcoxon test for paired samples was performed to compare pain at the different procedure time points during the first and second punctures. Pain assessed using the PIPP was correlated with the perception of pain reported by the health professionals using the Spearman correlation test. Data from the painful experience at time 3 (puncture) were used to perform the correlation because it was the most painful step of the procedure. The strengths of the correlations were analyzed considering values between 0.1 and 0.3 as a weak correlation, between 0.4 and 0.6 as a moderate correlation and above 0.7 as a strong correlation.^(14)^ A significance level of 5% (p < 0.05) was adopted in all analyses.

## RESULTS

The demographic and clinical data of the NB included in the study are provided in table 1. The health professional responsible for the punctures was primarily the laboratory technician (88.7%, n = 55). The number of puncture attempts to obtain successful sample collection ranged from one to seven punctures, and the median duration of the procedure was five minutes. During arterial puncture, a decrease in oxygen saturation was observed in 46.8% (n = 29) of the procedures. Changes in heart rate (bradycardia/tachycardia) occurred in 58.1% (n = 36) of the procedures, and in 56.5% (n = 35) of the observations, NB reacted with crying during and after the arterial puncture procedure.

**Table 1:** Characteristics of the newborns who underwent arterial puncture

Variable (n = 35)	
Days of life	18.5 (1 - 30)
Gestational age (full weeks)	34.0 (28 - 40)
Birth weight (g)	2,130 (885 - 3575)
Sex	
Male	22 (62.8)
Female	13 (37.2)
Main diagnoses	
Respiratory disorders	17 (48.6)
Major malformations	9 (25.7)
Sepsis	9 (25.7)
1-minute Apgar score	
0 - 4	5 (14.2)
5 - 7	22 (62.8)
8 - 10	8 (22.8)
5-minute Apgar score	
0 - 4	0
5 - 7	10 (28.5)
8 - 10	25 (71.4)

The results are presented as the median (minimum - maximum) or n (%).

Regarding pain assessment using the PIPP, no pain was identified in 30.6% (n = 19) of the observations. Mild and moderate pain was identified in 24.2% (n = 15) of the observations, and severe pain was identified in 45.2% (n = 28) of the observations.

The comparison of pain intensity between the degermation (T2) and puncture (T3) times in the first attempt showed that puncture was more painful than degermation. When comparing the intensity of pain between the first and second arterial puncture attempts, no significant difference was observed (Table 2).

**Table 2:** Comparison of pain intensity between the procedure time points (degermation and puncture) and between the first and second puncture attempts

	Median (min-max)	P 25	P 75	p value
Degermation	4 (0 - 14)	3.0	5.0	< 0.001
Puncture	9.50 (1 - 18)	5.0	14.25	
Puncture 1	9.50 (1 - 18)	5.0	14.25	0.007
Puncture 2	8.50 (3 - 18)	5.0	11.75	

p value < 0.05.

The correlation between pain assessment by the PIPP and the perception of pain reported by the health professionals was direct, and its intensity was moderate for the first puncture (rho = 0.533; p < 0.001) and strong for the second (rho = 0.824; p < 0.001) (Table 3).

**Table 3:** Spearman's correlation between the pain assessment by the researchers and the pain perception described by the professionals

Parameters	Rho	p value
Pain intensity P1	0.533	< 0.001
Pain intensity P2	0.824	< 0.001

p value < 0.05.

When the NB were stratified by gestational age (28 to 32 weeks, 32 to 36 weeks and above 36 weeks), no significant difference in pain intensity during arterial puncture was observed among the groups (p < 0.05).

## DISCUSSION

Newborns admitted to neonatal intensive care units are subjected to a large number of painful procedures during hospitalization, and there are many obstacles to the implementation of therapeutic measures in clinical practice.^(15,16)^

In the present study, the experience of pain in the investigated population ranged from absent to intense, a finding that demonstrates the specificities of the experience of pain among individuals, even when subjected to the same procedure. Based on the PIPP, the absence of pain was recorded in approximately one-third of the procedures, a finding that can be explained by the adoption of pain control protocols during arterial puncture, for example, the administration of a glucose solution and facilitated restraint. However, there was significant variation in pain intensity, a finding that indicates the need for an individualized approach when performing the procedure. The importance of critically evaluating the adoption of institutional protocols is also emphasized, as such protocols tend to propose the standardization of practices despite the specificities of individuals. This variation in pain intensity caused by arterial puncture reinforces the importance of adopting rigorous treatment measures as well as conducting a rigorous clinical evaluation of the appropriateness of the procedure.

Because arterial puncture is a short-term technique, exposure to manipulation occurs for a short period of time, explaining the absence of pain in the degermation phase and the marked presence of pain during puncture. Procedures that require more manipulation, especially in the degermation phase, such as the insertion of a central catheter by peripheral puncture and lumbar puncture, may be even more painful due to excessive manipulation, which points to the fact that the cortical interpretation of pain in NB may also be related to manipulation.^(17,18)^ During arterial puncture, in addition to technical skills and knowledge related to the anatomical and physiological conditions of NB, the health professionals involved in care must be able to perceive pain-related behavioral and physiological changes.^(19)^

The pain intensity during the first puncture attempt was greater than that during the second attempt. According to a review study by Valeri et al., early and repeated exposure to pain can promote neurological changes and impact the neurobehavioral responses of NB, resulting in a reduced pain threshold, hyperalgesia and allodynia stemming from the process of accommodation in cases of persistent pain.^(19)^ Exposing NB to two or more consecutive painful procedures may hinder the assessment of pain experiences in this age group.^(18)^ After successive exposures, it is expected that NB will no longer respond to the stimulus with exacerbated behavioral and physiological changes; importantly, this lack of response does not necessarily mean no pain.

Because it is a complex procedure that requires a theoretical background in anatomy and physiology and considering the current legislation on professional practice set forth in resolution no. 390/2011 of the Federal Council of Nursing (*Conselho Federal de Enfermagem* - Cofen), arterial puncture must be performed by professionals with training and technical skills. The main complications of arterial puncture are bleeding, bruising, peripheral ischemia and pain.^(20-22)^

The main diagnoses recorded among the NB included in this study were respiratory disorders, diagnoses that require a series of interventions for the maintenance of life in these patients. Several painful procedures are performed in NB admitted to the NPCU without analgesia, and it is important to note that this exposure can result in immediate and late complications, which can be identified during hospitalization or during child development.^(23)^

A study conducted with 26 NB admitted to a Brazilian neonatal intensive care unit compared the physiological parameters of NB before and during an arterial blood gas exam. It was found that 50% of the NB exhibited heart rate changes during the procedure, and 34.7% showed altered oxygen saturation (SatO_2_). However, although significant changes in heart rate and SatO_2_ were observed before and after the painful procedure, the use of these parameters alone is not sufficient to assess pain.^(18)^ This study corroborates our findings and reinforces the need to use physiological parameters always in combination with behavioral data to assess pain in the neonatal period.

Regarding crying, a study conducted in Brazil evaluated crying during venipuncture. The researchers reported that among the studied preterm NB who showed signs suggestive of absence of pain, 70% mumbled, whereas 30% did not cry.^(24)^ Crying is one of the manifestations of pain in NB, and although it is a sign that something is wrong, it may or may not be related to pain. Given the above, in the neonatal period, the experience of pain should be assessed using mixed methods, which include associated physiological and behavioral indicators.

The correlation between the assessment of pain intensity using the PIPP applied by the researchers and the perception of pain reported by the health professionals who performed the procedure was direct and moderate during the first puncture and direct and strong during the second puncture. This finding indicates that as the pain intensity increased, health professionals also perceived a greater pain intensity. Although pain perception is not a result of applying a validated scale, health professionals are able to identify painful experiences and, therefore, know that arterial puncture causes pain, a fact that should result in the adoption of therapeutic measures. A study conducted with 57 health professionals working in a neonatal intensive care unit in the interior of São Paulo showed that most were able to recognize when NB felt pain. However, many difficulties have been identified in relation to pain assessments and management, which can certainly be the cause of undertreatment in most neonatal units.^(25)^ A study conducted in Brazil with 24 health professionals who worked in neonatal units showed that 100% recognized that NB were capable of feeling pain; however, 58.4% were not aware of assessment scales.^(24)^ The literature is emphatic in recommending the use of validated methods for assessing pain experience, and there are many validated scales for assessing pain and sedation in the neonatal period,^(5)^ but even so, there are obstacles to the implementation of these instruments in clinical practice as tools for decision making.

One of the limitations of this study was the difficulty in monitoring sample collection because many were requested on an urgent basis and performed at times different than those established in the routine of the unit. Considering the scarcity of studies that assess pain during arterial puncture, the present study provides evidence for arterial puncture to be recognized as a procedure that requires rigorous pain control.

## CONCLUSION

Arterial puncture is a painful procedure for newborns, with intensities ranging from mild to severe. Pain is recognized by health professionals and identified through scales, which, therefore, should be used in the daily care routine in neonatal intensive care units as tools for treatment decisions. The results of this study confirm the findings in the literature about the importance of identifying physiological changes always in combination with behavioral changes. It is recommended that any procedure known to be painful to newborns should be performed considering some therapeutic approach, and pain should be evaluated during and after the procedure so that effective pain control is achieved. The results of this study contribute to the implementation of pain control strategies for newborns subjected to arterial puncture during hospitalization in neonatal units, reducing the immediate and late complications resulting from the performance of painful procedures without an adequate therapeutic approach.
